# Use of Saline Infusion Sonography to Evaluate Intrauterine and Tubal Factors in Subfertile Patients

**DOI:** 10.7759/cureus.73375

**Published:** 2024-11-10

**Authors:** Krishna Sailaja Sattiraju, Shriraj Katakdhond

**Affiliations:** 1 Obstetrics and Gynaecology, Dr. D. Y. Patil Medical College, Hospital and Research Centre, Pune, IND

**Keywords:** diagnostic techniques and procedures, fallopian tubal patency, female infertility, recurrent pregnancy loss, reproductive medicine, saline infusion sonography, uterine abnormalities

## Abstract

Objectives: This study aims to observe and report on the use of saline infusion sonography (SIS) to find out intrauterine and tubal factors in infertile/subfertile women, focusing on its diagnostic use, clinical advantages, and practical implications.

Methods: A prospective observational study was conducted involving 86 women presenting with subfertility and/or recurrent pregnancy loss in a tertiary care hospital. These participants were selected based on inclusion and exclusion criteria relevant to the study objectives. SIS was performed using B-mode ultrasonography and Doppler techniques, involving the instillation of saline transcervically into the cavity of the uterus. The procedure's use in evaluating uterine and tubal pathology was observed.

Results: SIS demonstrated reasonable diagnostic use in detecting uterine anomalies, including polyps and leiomyomas. The technique also assessed tubal patency, though pinpointing the exact site of obstruction was challenging. The procedure was well-tolerated, with minimal discomfort reported by the patients.

Limitations: The single-center design and limited sample size of the study are among its drawbacks, which could affect the generalizability of the findings of this research.

Conclusions: SIS is a valuable, minimally invasive diagnostic tool for assessing uterine and tubal factors in subfertile women. It offers high diagnostic value, low risk of complications, and improved patient comfort compared to traditional methods.

## Introduction

Infertility or subfertility (reduced fertility) is a growing concern in countries like India. According to the World Health Organization (WHO), approximately 17.5% of women globally face infertility issues [1]. As defined by the American College of Obstetricians and Gynecologists (ACOG), infertility is, “the inability to achieve a successful pregnancy after 12 months or more of regular unprotected intercourse” [2].

Tubal abnormalities play a major role in female subfertility, implicated in up to 40% of cases [3]. An essential first step in evaluating a subfertile couple is tubal factor screening. The primary tool for diagnosis and evaluation of patency of tubes and uterine anomalies is hysterosalpingography (HSG) despite its limitations, which include the use of iodinated contrast and X-ray exposure, which can cause discomfort and pain for patients. Laparoscopy, the gold standard for diagnosing tubal obstruction and early detection of conditions like endometriosis and pelvic adhesions, is invasive and carries risks such as nausea, shoulder tip pain, and injuries to the bowel, bladder, blood vessels, and ureters [4].

Saline infusion sonography (SIS) (or saline infusion sonohysterography), has gained acceptance as a method for screening in determining tubal patency in subfertile patients. SIS, the now widely used terminology, was first coined to describe this technique by Widrich et al. in 1996 [5,6]. They started the use of this term to better and more accurately denote the procedure, which was previously referred to as echohysteroscopy, the Parson’s technique, and artificial distension of the uterus using saline, among other descriptors. It involves the instillation of fluid, transcervically into the cavity of the uterus via a catheter, enhancing the visualization of the endometrium and assessment of tubes during transvaginal ultrasound (TVUS) examination [7]. SIS is recognized for its high tolerability, rapid execution, minimal expense, and low risk of adverse effects and complications.

Given the limited number of structured investigations to date, this research endeavors to consolidate current findings, identify areas for potential advancement, and contribute to ongoing efforts aimed at refining fertility assessment and improving reproductive outcomes for couples worldwide. This study aims to investigate the application of SIS in assessing tubal patency and endometrial factors among subfertile women, women with recurrent pregnancy loss, focusing on its diagnostic use, clinical advantages, and practical implications.

## Materials and methods

This was a prospective, observational study for a duration of eighteen months, from November 2022 to May 2024, involving subfertile women who presented for infertility evaluation at a tertiary care center, Dr. D. Y. Patil Medical College, Hospital and Research Centre, Pune, India. The study aimed to assess the utility of SIS in evaluating intrauterine and tubal factors. It was approved by the Institutional Ethics Sub Committee, Dr. D. Y. Patil Medical College, Hospital and Research Centre (approval number: IESC/PGS/2022/138) via a letter dated November 4, 2022.

Study population

The study included 86 subfertile women aged above 18 years, with a history of infertility, recurrent pregnancy loss, and secondary amenorrhea (pregnancy test was negative). Patients with known or suspected pregnancy, pelvic inflammatory disease, genital tuberculosis, severe endometriosis, unexplained active vaginal bleeding, or suspected malignancy were excluded. Post-menopausal and unmarried women were excluded. 

Procedure

**Figure 1:**
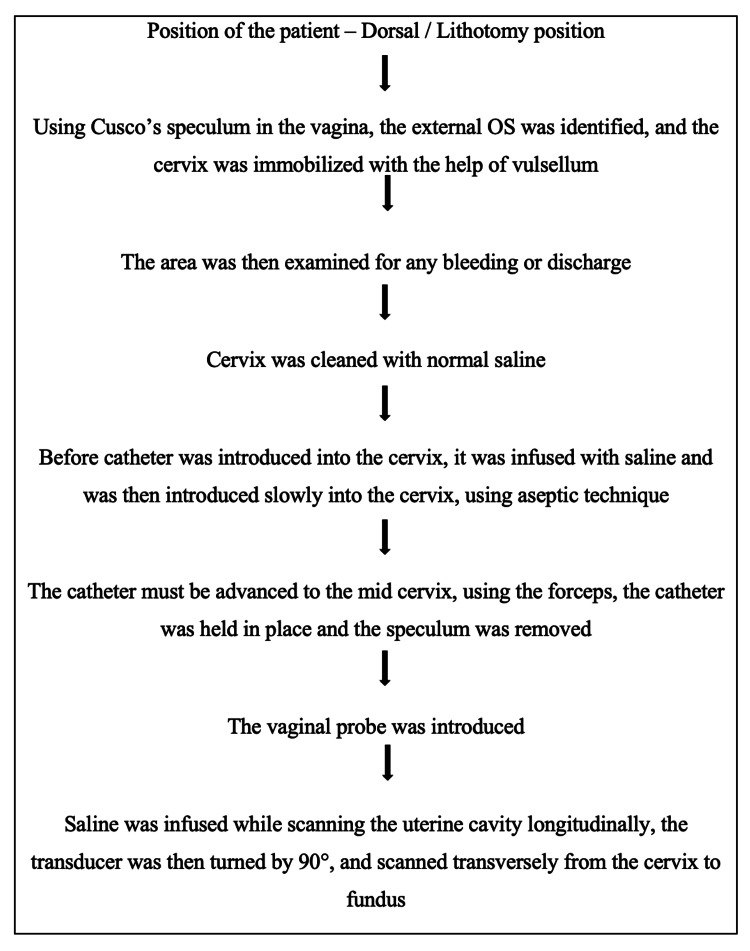
Step-by-step procedure of saline infusion sonography The flowchart was made by the authors on November 10, 2022

SIS was carried out during the proliferative phase of the menstrual cycle (day 7-10). A speculum was placed in the vagina, and with the use of an antiseptic solution, the cervix was cleaned. An 8 Fr Foley catheter was introduced into the cavity of the uterus, and saline was infused. TVUS was then carried out to visualize the cavity of the uterus and bilateral fallopian tubes as described in Figure 1.

Data collection

Data were collected on patient demographics, infertility duration, previous treatments, and SIS findings, including the presence of intrauterine pathologies (e.g., polyps, fibroids), tubal patency, and any complications.

Statistical analysis

Data was collected and entered in the predesigned Excel spreadsheet (Microsoft Corporation, Redmond, Washington, United States). IBM SPSS Statistics for Windows, Version 26.0 (Released 2019; IBM Corp., Armonk, New York, United States) was used to perform the statistical analysis. The study encompassed both qualitative and quantitative variables. The data was summarized with the use of descriptive statistics. Continuous variables were presented as mean, and categorical variables were presented as absolute numbers and percentages.

## Results

Our study included 86 participants, with primary infertility affecting 57% (49 participants) and secondary infertility affecting 43% (37 participants). Notably, there were 24 cases of primary infertility in the age group of 21-25 years and 22 cases of secondary infertility in the age group of 26-30 years, as shown in Table 1. 

**Table 1:** Age group and type of infertility of the SIS participants (N=86) SIS: saline infusion sonography

Age Group (years)	Type of Infertility		Total
	Primary, n	Secondary, n	
21-25	24	8	32, n
26-30	11	22	33
31-35	8	4	12
>36	6	3	9
Total	49	37	86

Individuals with primary infertility had a shorter mean duration of infertility (3.59 years) compared to those with secondary infertility (5.63 years), and their mean age was lower (25.98 years vs. 31.40 years). Among the 37 participants with secondary infertility, 23 reported a history of spontaneous abortions. The most common number of abortions per woman was three (43.6% of cases). 

SIS identified that 64 participants had patent tubes while the remaining 22 participants did not. In the primary infertility group, 73.5% (36 participants) had patent tubes and in the secondary infertility group, 75.7% (28 participants) had patent tubes. Among the 22 participants with blocked tubes, 13 belonged to the primary infertility group as represented in Table 2.

**Table 2:** Type of block and infertility among the SIS participants with tubal blocks (N=22) SIS: saline infusion sonography

Tubal block	Type of Infertility		Total, n
	Primary, n	Secondary, n	
Bilateral	4	4	8
Unilateral	9	5	14
Total	13	9	22

**Figure 2:**
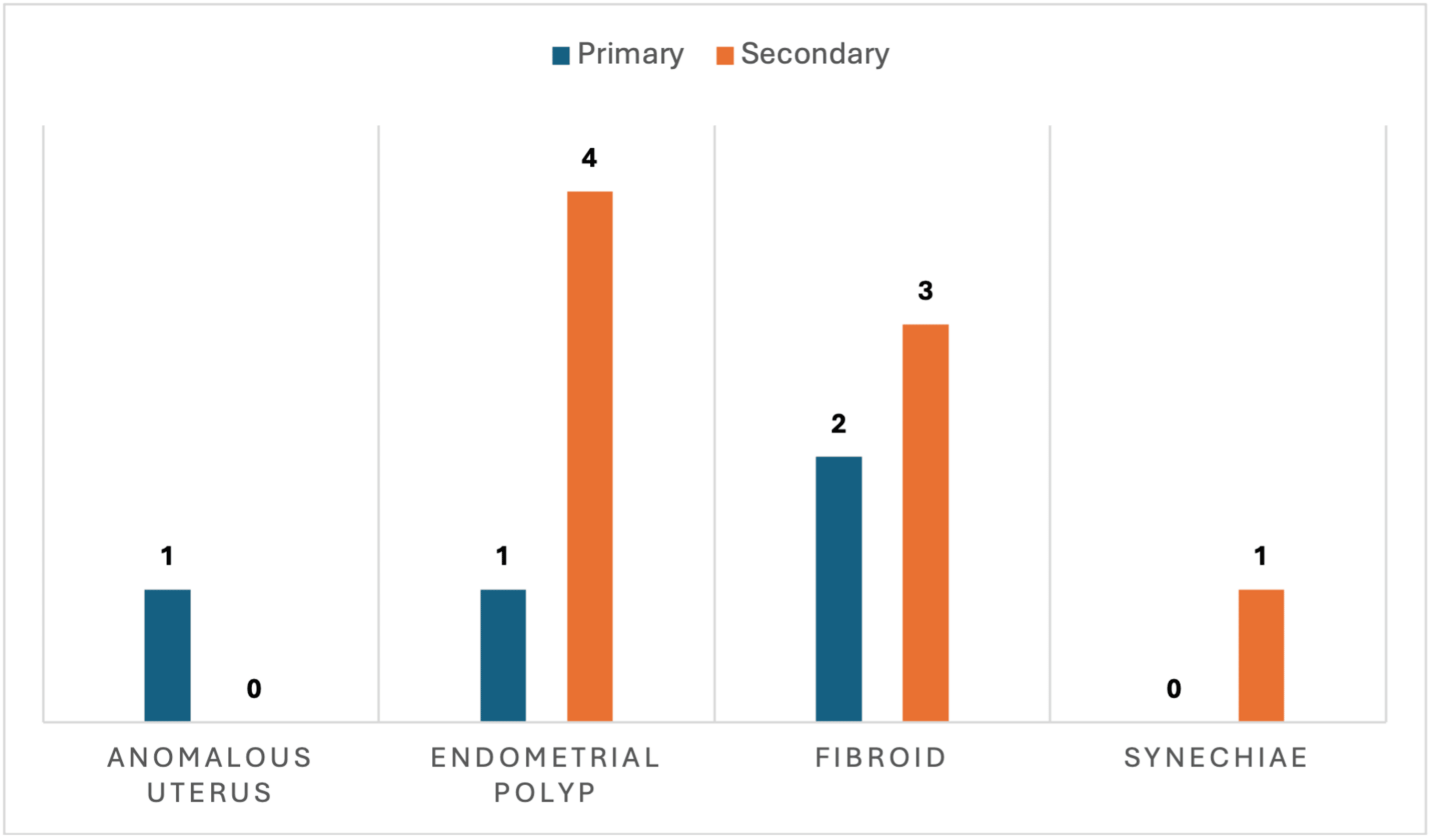
Types of filling defects with respect to type of infertility among the participants with intrauterine pathologies (N=12)

**Figure 3:**
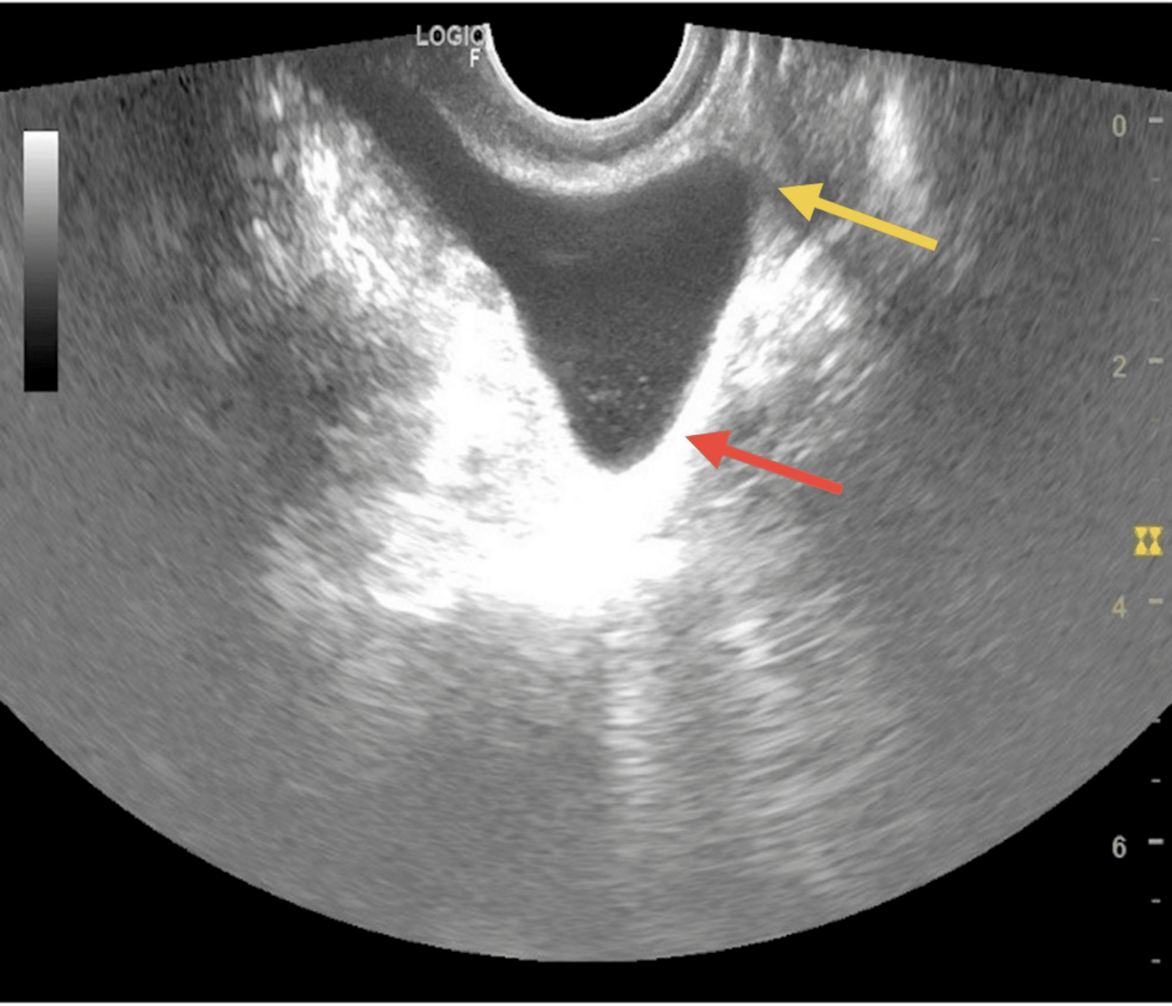
SIS showing unicornuate uterus SIS: saline infusion sonography Yellow arrow showing absence of cornua with tube unilaterally; Red arrow showing unicornuate uterine contour

Intrauterine pathologies in terms of filling defects in SIS were noted in 12 (13.9%) participants. Of these 12 participants, five had polyps, five fibroids, one anomalous uterus, and one had intrauterine adhesions (Figure 2). The one anomalous uterus was found to be unicornuate (Figure 3). The procedure was well-tolerated in general, with a mean pain score of 2.4 in the primary infertility group and 1.8 in the secondary infertility group and an overall mean pain score of 2.2 in the study participants on a 10-point scale.

## Discussion

The use of SIS in evaluating subfertile patients provides significant insights into uterine and tubal abnormalities. This discussion examines the findings within the broader context of existing literature, emphasizing the diagnostic use, clinical implications, and limitations of SIS.

In this study, 86 cases of subfertility were analyzed, with 57% being primary infertility and 43% secondary infertility. These results were comparable with Sitimani et al., who reported 56% primary infertility, while Özokçu et al. noted a higher percentage of primary infertility in their study at 88.5% [8,9]. The mean age and duration of infertility in our study were noted to be similar to other Indian studies [8,10]; however, it differed from that of Adenin et al., in which the population had a higher mean age and longer infertility duration [11]. This variation may be accounted for by etiological and demographic differences between the populations.

Recurrent pregnancy loss was observed in 26.7% of our study population, with 24.4% experiencing more than one abortion. This is comparable to the findings of Yigeremu et al. (38%) [12] and Moustafa et al. (16%) [13]. SIS findings showed 64 patent tubes and 22 blocked tubes in the current study, with results similar to Singh et al. [10] and El-Sayed Ali et al. [14]. SIS's role in evaluating tubal patency is crucial, offering a less invasive and more patient-friendly option compared to traditional methods like hysterosalpingogram. Strandell et al. demonstrated SIS's accuracy in assessing tubal patency with a good correlation to laparoscopic findings [15], unlike our study which was limited to the use of SIS and TVUS.

A similar study conducted by Moradan et al. showed only 8% positive findings in uterine abnormalities [16], which aligned with the findings of this study. In a study by Adenin et al., it was noted that there were no abnormalities in the uterine cavity in 94.2% of the cases [11]. SIS revealed endometrial polyps in 5.26% of the cases, synechiae in 0.36% of cases, and Müllerian duct anomalies in 0.18% of the cases, much like our study findings. However, the study's observational nature may have limited the study and significance and sensitivity could not be assessed.

SIS has proven superior to conventional methods like TVUS and HSG in identifying endometrial polyps, submucosal fibroids, and uterine adhesions. For instance, Soares et al. found that SIS had a sensitivity of 94% and a specificity of 88% for detecting intrauterine lesions, making it a reliable alternative to more invasive procedures like hysteroscopy [17]. Enhanced visualization with saline contrast significantly improves diagnostic accuracy as corroborated by studies such as those by Syrop and Sahakian [18] and Sitimani et al. [8]. 

Our study found no significant differences in pain scores between primary and secondary infertility groups, aligning with a similar study done by Ahmadi et al. [19]. In addition to this, complaints such as nausea, vomiting, or vaginal bleeding were not reported by a single patient during or after the procedure.

SIS is minimally invasive, cost-effective, and can be conducted in an outpatient format forgoing the use of anesthesia. It offers a comprehensive assessment of reproductive organs in a single session. In their study, Özokçu et al. suggested that SIS may be used as a “cost-effective” addition to the subfertility evaluation routine [9].

However, the procedure is not without limitations. Despite its advantages, SIS is highly operator-dependent, requiring significant expertise to interpret findings accurately. The quality of the TVUS equipment and the expertise of the operator can greatly influence the diagnostic outcomes. Some patients may experience discomfort or cramping during the saline infusion, which could limit the acceptability of the procedure. Standardization of training and protocols, as suggested by Brown et al., could help mitigate these issues and enhance the reliability of SIS [20].

The limitations of the study, besides the above, also include the small sample size, which although adequate for initial analysis, may not exemplify the broader population. Larger, multi-center studies could provide more definitive conclusions. Additionally, patient-reported pain scores, while valuable, are inherently subjective and can be influenced by various factors, including psychological state and previous pain experiences. Future research should focus on refining SIS techniques, integrating other imaging modalities, and conducting large-scale studies to compare long-term outcomes with traditional methods.

## Conclusions

SIS is an effective and valuable diagnostic tool for evaluating uterine and tubal factors in subfertile female patients. Its high diagnostic accuracy and cost-effectiveness, coupled with its non-invasive nature and minimal risk of complications, make it a valuable addition to infertility evaluations, and a preferred choice for many clinicians. Integrating SIS into clinical practice can enhance the assessment of subfertile women, ultimately improving reproductive outcomes. Further evidence is pivotal to validate the outcomes of this study, thoroughly understand the prospects of SIS in fertility assessments, and enhance its role in reproductive medicine.
